# "I do what I have to do to survive": An investigation into the perceptions, experiences and economic considerations of women engaged in sex work in Northern Namibia

**DOI:** 10.1186/1472-6874-11-35

**Published:** 2011-08-03

**Authors:** Alanna Fitzgerald-Husek, Alexandra LC Martiniuk, Reece Hinchcliff, Christine E Aochamus, Richard B Lee

**Affiliations:** 1 Michael G. DeGroote School of Medicine, McMaster University (Class of 2011), 1280 Main Street West, Hamilton, ON, L8S 4L8, Canada; 2 The George Institute for Global Health, PO Box M201 Missenden Road, Sydney, NSW, 2050, Australia; 3 Sydney Medical School, The University of Sydney, Edward Ford Building (A27), University of Sydney, Sydney, NSW, 2006, Australia; 4 Dalla Lana School of Public Health, University of Toronto, 155 College St,Toronto, ON, M5T 3M7, Canada; 5 Sunnybrook Health Sciences Centre, 2075 Bayview Ave, Toronto, ON, M4N 3M5, Canada; 6 Australian Institute of Health Innovation, The University of New South Wales, Level 1, AGSM Building, UNSW, Sydney, NSW, 2052, Australia; 7 The Namibian Business Coalition on AIDS, PO Box 25 746 Windhoek, Namibia; 8 The Department of Anthropology, University of Toronto, 19 Russell St, Toronto, ON, N5S 2S2, Canada

## Abstract

**Background:**

There is little published research investigating sex work in Namibia, particularly in rural areas. Therefore, the aim of this paper was to determine the views of women engaged in sex work in the Oshakati area of Namibia concerning the main factors influencing their use, or non-use, of male condoms during transactional sexual exchanges.

**Methods:**

Qualitative interviews were used to better understand the perceptions, experiences and economic considerations of female sex workers in Namibia who were involved in a Behavior Change Communication Program encouraging safer sex practices among high-risk populations in 2006 and 2007.

**Results:**

While the Behavior Change Communication Program has made significant strides in educating and empowering young women to negotiate more consistent condom use with sexual partners, the gendered economic inequalities and power imbalances within rural and semi-urban Namibian society that favor men hinder further advancement towards positive behavioral change for HIV prevention and also hinder the development of the loving relationships sought by some sex workers.

**Conclusion:**

This study found that sex workers and transactional sex encounters are heterogeneous entities dependent upon the characteristics of the man (known, stranger, wealthy, attractive to the woman) and the woman (in financial need, desiring love). These features all influence condom use. The 3 E's 'education, empowerment and economic independence' are critical factors needed to encourage and facilitate consistent condom use to prevent HIV transmission. Without financial independence and occupational alternatives building on their health education and empowerment, women who engage in sex work-and transactional sex more generally-will remain largely marginalized from Namibian society, and will continue engaging in risky sexual practices that facilitate HIV acquisition and transmission throughout the community.

## Background

Women who engage in forms of transactional sex, like sex work, often exemplify the considerable suffering and marginalization of women who lack economic independence [1]. As Campbell [2] notes, a hierarchically gendered social order systematically denies women, including those who engage in sex work, access to and control over economic resources and capital. Such gendered economic inequalities and related power imbalances place women who engage in sex work at considerable risk of contracting or spreading HIV/AIDS [3].

There is a high prevalence of HIV/AIDS throughout many low and middle income countries (LMICs), with heterosexual sexual relations primarily contributing to its spread. Educational and behavior change programs targeting high-risk individuals (e.g. female sex workers) and aiming to increase their use of male condoms have been implemented in efforts to prevent HIV transmission [4]. These programs have met with varying levels of success, with low rates of condom use amongst sex workers in many LMICs, including Namibia, identified in the limited available data [5,6]. It is likely that these programs do not effectively target the main factors influencing condom use, including the economic asymmetries, dominant patriarchal socio-cultural practices and resulting power differentials between men and women, all of which may influence the agency of women to actively and effectively negotiate safer sex practices, including condom use [7].

Power imbalances are particularly evident in rural and semi-urban settings such as Oshakati and the surrounding area in Namibia, where women typically experience more poverty, attain lower education levels, and have less prospects for employment in the formal economy than males [8,9]. The increasing importance of the cash economy in Namibia and reduced access for many females further marginalizes them, pushing them towards unstable livelihoods in the informal economy, and carrying dangerous implications for women's ability to protect their own health [10,11]. Previous research has examined the attitudes and experiences of sex workers in Ghana [12], the various forms and determinants of transactional sex in sub-Saharan Africa [1,13], and the types and efficacy of HIV transmission strategies targeting sex workers in South Africa and India [6], Mozambique [14], and elsewhere in the world [15,16]. The two studies conducted on sex work in Namibia are not in the peer-reviewed literature and have focused on the legalities of sex work in urban environments [17,18].

In collaboration with the Namibian National Ministry of Health and Social Services and several external funding bodies, numerous organizations are working to reduce the prevalence of HIV/AIDS within the country, which remains at approximately 17%, despite decreases over the previous decade [5]. One successful organization has adopted a multi-level approach to HIV/AIDS by addressing relevant individual, community and societal issues through a Behavior Change Communication (BCC) Program and a Condom Social Marketing Scheme. It endeavors to prevent HIV transmission by providing vulnerable groups with access to accurate HIV/AIDS-related information, and by ensuring the availability, accessibility and affordability of high-quality condoms [19].

This organization's BCC Program employs a KAB (knowledge, attitude, behavior) approach that emphasizes risk-reduction strategies. It operates in several high transit locations (e.g. larger centers along the major thoroughfares, and border towns/ports) and targets high-risk groups, such as female sex workers, including those in the Oshakati area, reflecting the increasing female focus of HIV/AIDS prevention strategies [20]. Community peer educators conduct weekly sessions on HIV/AIDS-related topics, such as the effectiveness of condoms in preventing the transmission of HIV. In addition, BCC peer educators encourage individuals to take ownership of their health and improve their health-related attitudes and behaviors.

This study sought to address gaps in the research and examine the perceptions, experiences and economic considerations of women who engage in informal sex work in a rural/semi-urban setting in northern Namibia. The aim was twofold: to assess the effects of the BCC Program on sex workers' ability to negotiate safer sexual practices, including condom use by clients and/or partners during transactional sexual exchanges; and to identify factors that may be limiting the effectiveness of the BCC Program. In doing so, this study sought to determine impediments to current HIV prevention programs in rural/semi-urban Namibia in reaching their aims and to propose recommendations for improvements to policy and programming to improve the health of women engaging in informal sex work and ultimately improve population health.

## Methods

### Recruitment of participants

Ten informal Namibian female sex workers (FSW) aged 17 to 24 years were recruited to participate in this study. Snowball and convenience sampling methods were used, as all participants were identified female sex workers already involved with the BCC program operating in three communities within a 35 km radius of Oshakati, Namibia.

The lead researcher observed and contributed to BCC discussions, which facilitated meeting and building rapport with sex workers in the region. Potential interviewees were approached by BCC peer educators and the lead author, who described the study and their potential role within it. The voluntary nature of the study was explained, along with the lack of compensation or rewards for their involvement. Consent was obtained verbally due to the low literacy levels amongst potential participants and existing cultural norms. The lead researcher assured confidentiality and participants' anonymity, and that all identifying details about participants would be changed.

### Methodology

A qualitative approach was employed to better understand the perceptions, experiences and economic considerations of women engaged in sex work in northern Namibia. In addition to BCC sessions, informal and scheduled interactions without BCC peer educators encouraged more open and active dialogue by all participants. These sessions increased participants' ownership over the study, offering them the opportunity to help determine the nature and direction of each meeting, including topics of discussion.

### Data collection and analysis

In addition to data gained through informal interactions in June-July 2006 and 2007, semi-structured interviews were also conducted with study participants. The general interview schedule was developed by the lead author in collaboration with the BCC Program Coordinator, and included questions about participant demographics, education, condom use, transactional sexual exchanges, financial status and personal aspirations.

Interviews were conducted in English, with peer educators and/or participants proficient in English providing translation into Oshiwambo, the dominant language of the region, as required. Written notes of the interviews were taken, with the resulting interview summaries stored in a secure location only accessible to the lead author. The data were thematically analyzed, with key issues iteratively developed and solidified into key categories. The results were discussed with the BCC Program Coordinator to verify their relevance and validity, then disseminated through two community forums held in July 2006 and July 2007. This project received appropriate ethical consideration by the University of Toronto Research Ethics Board and was approved by NaSoMa prior to commencement.

## Results

Information about participants' demographics, lives and experiences emerged within several themes from the research, including participants' reasons for engaging in sex work, sexual beliefs and practices, perceptions of the BCC Program, and barriers to increased condom use during transactional sexual exchanges.

### Participants' profile and demographics

All ten participants were born in different parts of Namibia within varying ethnic groups, and now lived within 35 km of Oshakati, in areas where the BCC program had been operating for over a year. While some individuals travelled to neighbouring towns along the main road, the informants generally were not very mobile, and some resided in the same accommodation. The majority of informants had been engaged in informal sex work for three to six years, and differentiated between themselves and more commercial sex workers in larger urban centres, *"I know of them, but they are not like us. These [commercial sex workers] are women who go to Windhoek and Swakop[mund] and Walvis Bay and are looking for men, rich Namibians or maybe tourists. They are different from us." *Informants' partners/clients were from the local community, and were often met in local drinking establishments, where alcohol (but no drugs) was often involved. Two had children (one woman's child was still in her care), and one acknowledged that she was in a relationship with a "long-time boyfriend" outside of transactional sexual partners/clients. Participants had limited formal education, having left school between grades four and eight for various reasons including poverty and family circumstances. One informant explained that the death of her mother forced her to leave school at age thirteen and abandon her childhood aspiration to become a lawyer.

### Reasons for engaging in sex work

The majority of informants described themselves as unemployed with no regular source of income, and lacking skills with which they could secure an alternate source of income. This forces a reliance on men for food and other necessities. As one participant noted, *"when I'm needing something, maybe needing some money for food, I will go and get it from a guy [in exchange for sex]"*. It was further noted that such exchanges can also involve the acquisition of new clothes and other commodities that may facilitate increasingly profitable future sexual exchanges. As another informant noted, *"girls who look better get better money, because men think these girls are not simple things and they are used to getting more"*.

Sex workers who were interviewed expressed frustration at their current activities, with one informant stating, *"I am sick of this! I hate this... I hate this, what I have to do to survive. I am tired of this [of being a sex worker]...I don't want to do this any longer. I want a way out. I want a better life"*. Indeed, the general rationale provided by informants to explain their engagement in sex work was summarized by one woman who explained, *"I do what I have to do to survive. It is the same with many of the others here"*.

### Sexual beliefs and practices

While their involvement in the BCC Program increased many participants' overall understanding of sexual health issues, there were still many misconceptions regarding HIV and pregnancy. For example, several informants did not know HIV transmission is influenced by sexual health status and sexually transmitted infections (STIs), and thought it was impossible to become pregnant if one only engaged in unprotected sex while menstruating. Indeed, the fear of falling pregnant was the most prominent issue raised by informants, with HIV transmission identified as a lower priority. In explaining the reason for this disparity, the visible nature of pregnancy was particularly emphasized, with one informant noting, *"if I fall pregnant, everyone will see, but if I am having HIV, maybe you cannot see it if I am not sick"*. Indeed, a common strategy for negotiating condom usage was promoting its use for contraception purposes rather than preventing HIV transmission, as it was felt that men may be more agreeable to use condoms if suggested as a barrier for preventing unwanted pregnancy, as opposed to HIV.

### Organizational activities

Despite holding some incorrect assumptions about HIV and pregnancy, informants nonetheless appreciated and trusted the information received through the BCC Program. As one young woman noted, *"[It] teaches us about HIV and such things that we didn't really know or understand before. It helps us know the risks, and why using a condom is good, and many other things"*.

Participants felt that regular interpersonal, interactive sessions-as opposed to pamphlets or television messages-were the most valuable method of communication. The trust and respect awarded to peer educators, as opposed to family members or hospital staff, was identified as the main reason for the effectiveness of these interpersonal interactions, with a sex worker stating, *"some parents don't talk about this [HIV, condom use] and may be asking 'what is this?' if you have or are asking about sex and condoms. So people need to learn these things from peer educators who know about them"*. Indeed, participants noted that there were few non-judgmental environments in Namibian society besides BCC sessions in which open dialogue regarding HIV transmission and condom use may occur.

In addition to disseminating information, the BCC peer educators aim to empower female sex workers to actively negotiate sexual decision-making. BCC activities included both female-only and co-ed sessions in gender-neutral spaces, with question/answer opportunities, condom use demonstrations, role-playing exercises, and attempts to disband myths and stereotypes. Programs which more overtly promoted women's empowerment commonly met with negative responses from men within the community, who often viewed such strategies as attempts to reduce their own status and power. As one peer educator explained, *"if men see we are helping women have a voice, they will maybe get angry and think that women are taking away their power"*.

Due to the efforts within the BCC Program to disseminate health information and promote female empowerment, participants commonly described it as a success, emphasizing its positive effect on their attitudes towards their rights, health and sexual practices. As one informant explained, *"other girls who haven't learned this kind of information don't know that they have the right to protect themselves"*.

Participants further explained that the BCC Program had increased their sense of love, respect and protection towards their bodies, which increased their attempts to negotiate condom use with male partners/clients. Indeed, one sex worker explained that due to this newfound bodily respect and empowerment, she felt she had *"a right to insist on using a condom... If I ask him to use [a] condom then he should agree to it, and if he doesn't, you know there is something wrong with him: maybe he is sick, or has HIV, or is hiding something. He should respect himself enough to want to use [a] condom, otherwise I shouldn't agree to have sex with him"*. In summarizing the common view of the generally positive effect of the BCC Program, one participant noted, *"now we're more afraid, but it is a good thing. We take better care of ourselves and use condoms"*.

### Barriers to engagement in safer sexual practices

Despite an overall increase in condom use since becoming involved with the BCC Program's efforts, participants stated that at best, condoms were now used in six out of every ten transactional sexual encounters, with some stating that they still rarely insisted on condom use, The sex workers who were interviewed explained that inconsistent and/or lower condom use rates could be attributed to numerous complex, interconnected economic and socio-cultural factors. Despite their involvement in the BCC Program and feeling more empowered, these women largely lacked the power to make sexual decisions, such as negotiating condom use, as this was widely viewed as the domain of males, on whom they were economically dependent. Additionally, sex workers' attempts at negotiating safer sex practices were often met with men's widespread resistance to condom use and perception of condoms as a threat to their masculinity. In this sense, condom use was seen to be associated with infidelity, promiscuity, and a lack of trust between partners. An additional issue voiced by informants was that men often viewed sex with a condom as unnatural and unpleasant.

Condom use also varied both between sex workers, and amongst one woman's partners, depending on her attitudes towards and relationship with a particular partner/client. A number of informants endorsed that it was easier to insist on condom use with new or "one-off" partners, as opposed to repeat or more regular clients, for reasons such as fear of losing that partner or source of income. Similarly, the participant in a long-term relationship commented, *"it's harder to insist on using [a] condom with my boyfriend than a stranger"*.

An additional reason for lack of condom use expressed by informants was the promise of obtaining love and a better life, with several describing incidents where male sexual partners promised ongoing love and care. As one sex worker noted, *"maybe if you refuse to have sex without [a] condom like he wants then he won't look after you and will leave. It is hard to say no if you maybe get a nice life and family with him"*. Another informant similarly described the appeal of becoming pregnant with a handsome or wealthy man's child: *"in this way, other people will see you and know that it is his child. He is your man and you are his [woman]"*.

Nonetheless, sex workers voicing this perspective were not naïve, and were still afraid of becoming pregnant. Many were acutely aware of examples where men had promised love and a better life but then left the woman once she became pregnant. However, the hope and promise of provisions of love, a family, money and material resources often outweighed participants' desire or insistence on condom use to prevent possible HIV transmission and potentially unwanted pregnancies. Such views indicate the association between economic priorities and sexual practices, including condom use, amongst Namibian sex workers. Indeed, informants frequently explained that they commonly prioritized their immediate financial requirements over less urgent problems that potentially had severe, future implications, including HIV infection. Oral contraceptives and other family planning methods were not commonly used among those interviewed.

Due to some male partner's views that women who insist on condoms must *"be dirty or have HIV"*, as well as men's ability to determine the price and conditions of sexual activities, the prioritization of economic needs often resulted in sex workers being coerced to engage in risky sexual behaviors. As one informant explained, *"if I try to insist on using condom[s] during sex, he may give me less money or decide not to go with me at all. And I am needing the money. So this means that sometimes I must go with him and have unprotected sex"*. Indeed, another sex worker noted that too much insistence on condom use may *"make the guy go with another girl instead"*, which often results in their need to engage in future possibly riskier sexual exchanges for less money.

## Discussion

Our research has revealed several characteristics and experiences of women who engage in sex work in rural Namibia which help to understand the limitations of the effectiveness of the BCC program. Our research found that the critical factors related to having protected sex varied in different circumstances. For instance, when a FSW was low on cash, financial factors may be critical. If the FSW was attracted to the man, or if he was rich, or if she hoped to become his girlfriend or have his baby then she was more likely to have unprotected sex. These research findings are thus novel for this setting in that they go beyond thinking about increasing condom use as driven by education and even beyond the newer thinking of the 3Es: education, empowerment and economic independence to think about transactional sex as heterogeneous and thus driven by different factors. In our study FSW were driven by the desire for money or love or both, and this depended on the types of male partner (primary, regular, stranger), the conditions of the exchange (pre-determined, a gift) and on the socio-economic status of the man (local, business man, tourist).

In our study the participants were less commercialized or formalized as sex workers compared to urban settings. These informal FSWs did not consistently define themselves as a sex worker but instead engaged in transactional sex under certain circumstances. Since many HIV prevention programs target more formalized sex workers, the findings from our study have important policy implications as they demonstrate that in rural Namibia sex workers are not always a clear, defined group and transactional sex may be being used by much larger proportions of the population thereby increasing HIV transmission risk in the population. This underscores the need to move preventative interventions typically offered in a venue such as a brothel and targeted toward FSW to become more diffuse and reach out to the broader population of women who may not typically define themselves as a sex worker.

Due to the high rate of HIV/AIDS infection in Namibia, research, risk reduction programs and policy initiatives are required to combat HIV/AIDS in high-risk populations, such as women engaging in sex work, including those involved in this study. Such initiatives, like the BCC Program, commonly employ a knowledge, attitude, behavior (KAB) approach, whereby the acquisition of knowledge (e.g. correct and consistent condom use can prevent HIV transmission) should result in attitude adjustment (e.g. intention to use condoms), which should ultimately produce behavior change (e.g. consistent and correct condom use) [21]. While this approach is likely helping to curb the HIV prevalence rates in many countries [5], the KAB model does not in itself adequately address the full range of factors influencing behavior change, and thus further comprehensive prevention strategies are warranted, particularly where HIV persists in vulnerable populations [18].

In particular, the KAB model often oversimplifies or ignores other internal and external forces impacting upon behavior change, such as financial and socio-cultural pressures. As this study demonstrates, knowledge of safe sex practices and the intention to use condoms do not necessarily result in consistent condom use for women engaged in transactional sexual activities. Condom use was also found to be influenced by sex workers' perceived control over their own sexual decision-making processes [22], which is tied to economic and socio-cultural constraints. This finding is consistent with studies of female sex workers in urban environments in Namibia [17], in other LMIC, Nigeria [13], Ghana [12], the Caribbean [23] as well as in high income countries, the USA [24] and Canada [25] but may, arguably, be even more heightened in rural, traditional environments like northern Namibia. As such safer sex for females engaging in transactional sexual practices-and Namibian women, in general-may be more accurately viewed as a product of the '3 Es' of education, empowerment, and economic independence. Our study revealed further nuances beyond the emerging acknowledgement of the importance of 3 Es. The desire to be loved may encourage FSW to consent to high risk sexual practices. There was a desire by some FSW to become a certain man's girlfriend/wife and this may involve having a child, in part because FSWs say "having his kid shows that you're his woman". Further, FSW talked about the social capital of having a wealthy man's child so much so that even if the man leaves the woman may be considered to be of higher social standing.

From this new knowledge we have gained further insight into the complex factors likely required to prevent HIV transmission in this population of FSW and thus potentially slow the epidemic throughout populations. For women who are similar to the FSW in this study, the KAB model is likely a useful beginning (education, condom distribution, voluntary counselling and testing) but even more important are the 3 Es (education, empowerment and economic independence) and potentially of utmost importance, gender equality and the opportunity to develop equal and loving relationships. To these ends therefore, recommendations for ways forward for individuals similar to the women included in this study include addressing gendered inequalities- economic and otherwise, that are maintained by patriarchal socio-cultural practices. Men and women, including community leaders, businesses and government should be encouraged to better represent women [26]. This supports the previous recognition in Namibia that there is a need "to work toward elevating the perceptions of women in society through education institutions as well as political and traditional leaders" [3].

One specific strategy suggested by previous research in India and South Africa to empower and educate female sex workers is to continue training and encouraging them to become peer educators themselves [6]. Although on the other hand, a peer educators approach in Namibia or elsewhere may not be helpful since it is unlikely to impact upon the economic needs for sex work-being a peer educator is rarely a paid role. However, it is possible that a peer educators approach could address other features of sex work in this setting, such as engaging in sex work with the aim to develop a loving relationship. Such a peer educator program however, will need to be trialled in future research to assess the program's content and approach and to evaluate its efficacy and acceptability. As well, the women in our study pointed out that community members negatively view programs aimed at empowering women, to the extent that a peer educator stated that men may become 'angry' if they feel a program is doing this. Thus, in contexts where such programs may be found threatening by men, future empowerment programs will need to take into account the safety of participating individuals.

Our research found that sex work did not necessarily provide the financial independence sought by the women. Men in these circumstances still set the rules, the prices and the conditions of the exchange and thus the women remained economically dependent on the male. This underscores the importance of facilitating females' economic independence through vocational training opportunities for interested sex workers, and developing income-generation projects and individual and group micro-credit programs targeting these women. As a result of the research findings reported in this paper which were circulated in 2007 as an internal report within the local Namibian NGO, programs to address these needs have recently been initiated. Coupled with developing and supporting the circumstances in which female sex workers could utilize their new skills and knowledge, this may allow these women to become more financially independent and potentially obtain a viable economic alternative to sex work. This increased economic security may further empower women, adding to their ability to negotiate safer sexual practices, which, in turn, reduces the risk of HIV acquisition and transmission [13,17]. Since the recognition of the importance of economic security in relation to HIV prevention is relatively new there are few projects that have evaluated this approach in Namibia or elsewhere. For instance a 2004 Cochrane review [27] and a 2002 Lancet systematic review [28] of HIV prevention programs do not include evaluations of structural or economic programs. Though a 2009 publication describes an evaluation of the Sonagachi Project for Indian sex workers which indicates that broader strategies like social supports, advocacy, and micro-finance can significantly reduce vulnerability to HIV compared to more narrow approaches of clinical and education services alone [29].

There were several limitations involved in this study, including language difficulties and translations between English and Oshiwambo. However it is not anticipated that language challenges had a large impact on data acquisition or interpretation since BCC Program staff and other informants proficient in English acted as translators.

A second limitation is the potential that results have been affected by social desirability bias. Participants could have provided answers that they believed the interviewer desired. To avoid this potential problem, the interviewer attempted to remain open-minded and unbiased, avoided leading questions and organized interactions and interviews with participants without the presence of BCC Program representatives.

Finally, this study was restricted to exploring the views of informal female sex workers regarding their transactional sexual activities, including their use of condoms. The new knowledge gained from exploring the views of female sex workers in this study may not apply to all FSW. A range of other critical issues that emerged from the fieldwork were not pursued, including types of male partners (e.g. primary, non-primary/transactional, regular, one-time), conditions of exchange (e.g. money/"gift" given at every encounter, predetermined or variable amount), and socio-economic status of transactional partners (e.g. local man, businessman, tourist). Further analyses of these issues may similarly highlight qualitative information potentially important for HIV prevention strategies, and policy implications.

## Conclusion

As women engaged in sex work-and other young women-in Namibia and elsewhere continue to be affected and infected by HIV, the voices of this marginalized social group need to be heard and used to affect policy decisions with implications for their health, welfare, rights and livelihoods. Through this exploratory study aiming to elucidate and highlight the experiences and views of informal female sex workers in the Oshakati area of Namibia, it emerged that a KAB approach alone is insufficient to achieve safer sexual practices, including regular condom use. Women in this study demonstrated that FSW in rural Namibia are not a homogeneous group and not always defined by their sex work. Therefore, a "one size fits all" approach to HIV prevention is unlikely to work. The informality of their sex work underscores the importance of addressing sex work as an important aspect of the overall HIV epidemic in communities. The women in this study highlighted new features related to their use or non-use of condoms including economic but also importantly it was highlighted that condoms are not used when there is the desire to develop a loving relationship with the male. Thus, the KAB model oversimplifies and potentially ignores important factors influencing behavior change, such as existing gendered economic and socio-cultural asymmetries and resulting power differentials. It is primarily due to these factors that women engaging in transactional sexual activities are largely unable to determine the price and conditions of sexual exchanges, including consistent condom use. As well, some women did not aim to use condoms as they hoped the transaction would evolve into a 'real', loving relationship. Information dissemination, open dialogue, and attitude adjustment are important. This study found similarities to recent research with FSW in other countries showing the necessity of empowering females and promoting their economic independence in order to address the HIV epidemic. This study also observed a factor not typically discussed in the literature that in some circumstances FSWs' do not use condoms because they hope the transaction will evolve into a longer-term, loving relationship. Education about condom use is certainly not enough. We need to better understand the interface between condom use or non-use and the development of loving relationships in order to incorporate this into revised strategies for promoting sexual health. We need to empower women to protect their own health but also we need to foster deeper structural change equalizing the status of women in society and thus provide the supporting environment for women who desire to build a loving relationship as well as gain access to skills/training and access to the formal economy rather than engage in sex work. A focus on sex workers is important in its own right as these individuals are some of the most marginalized in society but also because sex workers comprise a focal point for the HIV epidemic and thus protecting the health of sex workers in one way to curb the HIV epidemic. Future research which evaluates the ability of larger, societal or structural changes to reduce risk behaviors for HIV transmission in FSW will be a useful next step.

## Competing interests

The authors declare that they have no competing interests.

## Authors' contributions

AFH conceived of the study, and participated in its design and coordination and helped to draft the manuscript. CEA helped conceive of and coordinate the study, and assisted with the data analysis. RH and AM assisted with the data analysis and wrote the manuscript. All authors read and approved the final manuscript.

## Pre-publication history

The pre-publication history for this paper can be accessed here:

http://www.biomedcentral.com/1472-6874/11/35/prepub
